# Research progress of glutathione peroxidase family (GPX) in redoxidation

**DOI:** 10.3389/fphar.2023.1147414

**Published:** 2023-03-02

**Authors:** Jun Pei, Xingyu Pan, Guanghui Wei, Yi Hua

**Affiliations:** ^1^ Department of Urology, Children’s Hospital Affiliated to Chongqing Medical University, Chongqing, China; ^2^ Chongqing Key Laboratory of Children Urogenital Development and Tissue Engineering, Chongqing, China; ^3^ Department of Pediatric Surgrey, Guizhou Provincial People’s Hospital, Guiyang, China

**Keywords:** glutathione peroxidase, redox balance, oxidative stress, inflammation, iron death

## Abstract

Maintaining the balance of a cell’s redox function is key to determining cell fate. In the critical redox system of mammalian cells, glutathione peroxidase (GPX) is the most prominent family of proteins with a multifaceted function that affects almost all cellular processes. A total of eight members of the GPX family are currently found, namely GPX1-GPX8. They have long been used as antioxidant enzymes to play an important role in combating oxidative stress and maintaining redox balance. However, each member of the GPX family has a different mechanism of action and site of action in maintaining redox balance. GPX1-4 and GPX6 use selenocysteine as the active center to catalyze the reduction of H_2_O_2_ or organic hydroperoxides to water or corresponding alcohols, thereby reducing their toxicity and maintaining redox balance. In addition to reducing H_2_O_2_ and small molecule hydroperoxides, GPX4 is also capable of reducing complex lipid compounds. It is the only enzyme in the GPX family that directly reduces and destroys lipid hydroperoxides. The active sites of GPX5 and GPX7-GPX8 do not contain selenium cysteine (Secys), but instead, have cysteine residues (Cys) as their active sites. GPX5 is mainly expressed in epididymal tissue and plays a role in protecting sperm from oxidative stress. Both enzymes, GPX7 and GPX8, are located in the endoplasmic reticulum and are necessary enzymes involved in the oxidative folding of endoplasmic reticulum proteins, and GPX8 also plays an important role in the regulation of Ca^2+^ in the endoplasmic reticulum. With an in-depth understanding of the role of the GPX family members in health and disease development, redox balance has become the functional core of GPX family, in order to further clarify the expression and regulatory mechanism of each member in the redox process, we reviewed GPX family members separately.

## 1 Introduction

The glutathione peroxidase family (GPX) is a family of antioxidant enzymes that are an essential class of selenoenzymes in mammals (Zhao et al., 2019). They belong to the same class of heme-free thiol peroxidases as peroxidases and catalyze the reduction of H_2_O_2_ or organic hydroperoxides to water or corresponding alcohols, thereby mitigating their toxicity (Zhao et al., 2019). Selenium cysteine (Sec) is the catalytic activity neutral of GPX, and its activity is closely related to the level of selenium content in the body. There are eight types of GPX family in mammals (GPX1, GPX2, GPX3, GPX4, GPX5, GPX6, GPX7, GPX8), of which GPX1-4 and GPX6 of mammals are considered to be selenoproteins containing selenium, which have antioxidant functions, and GPX6 is only expressed in the human body (Brigelius-Flohé and Maiorino, 2013; Barbosa et al., 2017).

In mammals, GPX works with superoxide dismutase and catalase to form an enzymatic antioxidant system that reduces reactive oxygen species (ROS) and limits their toxicity. For decades, GPX typically uses glutathione (GSH) as a reducing agent in catalyzing the reduction of H_2_O_2_ or organic hydrogen peroxide to water or corresponding alcohols, respectively (Brigelius-Flohé and Maiorino, 2013). The presence of sec as a catalytic component can guarantee a rapid reaction with H_2_O_2_ and rapid reduction by GSH (Handy et al., 2021). GPX-like proteins have been reported in many species, the importance of Sec has been recognized, and Sec-based glutathione peroxidase has been extensively studied in mammals, but they appear to play different roles in different locations or cells (Handy et al., 2021). GPX1 is ubiquitous in cytoplasm and mitochondria, formerly known as cytoplasmic GPX (CGPX), is the first discovered member of the GPx family, almost all cell mitochondria and cytoplasm can be detected, it is also one of the most widely expressed and highest content of selenoproteins in the human body (Alehagen et al., 2021), its main role is to catalyze glutathione (GSH) to reduce toxic peroxides in the body, including hydrogen peroxide, cholesterol peroxide and long-chain fatty acid peroxide (Alehagen et al., 2021; Handy and Loscalzo, 2022). The GPX2 is mainly expressed in the gastrointestinal system, including esophageal epithelium, intestinal epithelial cells, etc., and studies have shown that GPX2 can be used as the first line of defense against oxidative stress from food or intestinal flora. Its catalytic substrates include hydrogen peroxide, tert-butanol peroxide, etc. (Florian et al., 2001). GPX3 is predominantly present in plasma (Zhao et al., 2019). Its mature protein is mainly produced by renal tubular epithelial cells and subsequently secreted into the bloodstream, which may become the first barrier involved in mediating redox balance (Zhao et al., 2019; Chang et al., 2020). GPX4 mainly protects cell membranes from oxidative attack, and studies have pointed out that there are three different subtypes, namely sperm karyotype, mitochondrial type and cytoplasmic type, which can catalyze complex lipid hydroperoxides such as phospholipids, cholesterol, and cholesterol esters in addition to H_2_O_2_ (Bersuker et al., 2019). GPX5 is described in the epididymis as a secreted protein that contains cysteine (Cys) in the active center instead of Sec, GPX5 is the first discovered Cys-based superfamily member, also known as epididymal GPX (EGPX), which has now been shown to play a role in protecting mouse sperm from hydrogen peroxide toxicity (Zhang et al., 2008; Luan et al., 2019). In humans, GPX6 is a selenium protein with Sec as the active center. However, in rats or mice with Cys as the active center, it is mainly expressed in olfactory epithelial cells of rats or mice and was previously named olfactory metabolic protein (OMP) (Brigelius-Flohé and Maiorino, 2013). GPX7 and GPX8 were the last members to be discovered in mammals based on Cys, and were found to have low glutathione peroxidase activity. GPX7 may improve non-alcoholic steatohepatitis by regulating oxidative stress levels (Wei et al., 2012). GPX8 can inhibit the oxidative stress response of hepatocellular carcinoma cells (Lee et al., 2021).

A great deal of research on the structure and function of GPX in mammals has helped us understand the essential functions of this class of proteins. However, there is currently a lack of systematic research on the role of the entire GPX family in oxidative stress responses. To date, few studies have desystematized the role of GPX in oxidative stress. This study aims to explore the relationship between GPX and oxidative stress in mammals, and provide new ideas for alleviating oxidative stress in the future.

## 2 GPX family and oxidative stress

### 2.1 GPX1

**FIGURE 1:**
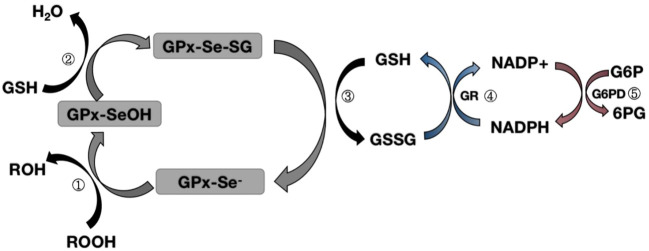
Map of the mechanism of glutathione peroxidase redox pathway.Step ①: Selenol (-Se^-^) in GPX is oxidized to selenic acid (-Se-OH) by peroxide (ROOH);Step ②: The first GSH molecule reduces selenic acid (-Se-OH) to form glutathioneated selenol intermediate (-Se-SG) and releases a part of H_2_O; Step ③: The second GSH molecule continues to reduce the intermediate (Se-SG) to form oxidized glutathione (GSSG), while the activity of GPX neutrality returns to selenol (-Se^-^);Step ④: Oxidized glutathione (GSSG) is degraded to reduced glutathione (GSH) under the action of NADPH-dependent glutathione reductase (GR), while NADPH loses an electron to NAPD^+^.Step ⑤: Glucose 6 phosphate (G6P) reduces NAPD^+^ to NADPH when circulating under the action of glucose 6 phosphate dehydrogenase (G6PD).

Glutathione peroxidase 1 (GPX1) was first identified in 1957 as a red blood cell enzyme that protects hemoglobin from oxidative stress (MILLS, 1957). Subsequent studies have found that the mineral selenium is essential for GPX1 activity (Seale et al., 2020). GPX1 is a tetramer that can react with H_2_O_2_ and soluble low-molecular hydroperoxides, but not with more complex lipid hydroperoxides. During the reduction of peroxidase by mammalian GPX1, the Sec active site needs to be modified to form an intermediate stable form (Kraus et al., 1980; Trenz et al., 2021; Flohé et al., 2022). After the reaction with peroxide, the original selenol (SE-H) active site is replaced by selenic acid (SE-OH) (Kraus et al., 1980; Trenz et al., 2021; Flohé et al., 2022). At this time, a GSH molecule is required to reduce selenic acid to produce glutathioneated selenol (SE-SG) intermediate (Kraus et al., 1980; Trenz et al., 2021; Flohé et al., 2022). At the same time, the second GSH molecule continues to reduce the SE-SG intermediate, forming oxidized glutathione (GSSG), and leading to the recovery of the active site (Kraus et al., 1980; Trenz et al., 2021; Flohé et al., 2022). The subsequent degradation of GSSG involves the action of NADPH-dependent glutathione reductase, which in turn interconnects GSH with the G6PD cycle to form a complete oxidation-reduction pathway (Kraus et al., 1980; Trenz et al., 2021; Flohé et al., 2022) (Figure 1). Since GPX1 has a certain reducing ability to hydroperoxides, it is classified as an oxidative stressase. GPX1 KO mice developed normally, indicating that the lack of antioxidant defenses could be compensated for by other proteins, or that oxidative stress under “normal” conditions did not cause any harm. Conversely, severe acute oxidative stress killed GPX1−/− mice, while wild-type mice supplemented with selenium survived normally. These findings clearly show that GPX1 is irreplaceable by other selenoproteins in protecting against systemic oxidative stress, and GPX1 has a significant antioxidant function in the body (Liu et al., 2021).

On the surface, the mice after GPX1 KO performed normally, but these mice were susceptible to the production of oxidative substances, and when stimulated, they were easy to cause harm to mice and increase oxidative stress *in vivo*. In many *in vivo* disease studies or oxidative stress stimulation models, GPX1 deficiency enhances cell damage, apoptosis, and even leads to cell death. For example, in the study of traumatic brain injury in mice, the lack of GPX1 increased the attack of ROS on hippocampal neurons, causing damage to post-traumatic memory function (Kim et al., 2022). In studies of myocardial ischemia-reperfusion injury, GPX1 KO exacerbated oxidative stress injury during myocardial ischemia-reperfusion (Lu et al., 2022). In addition, in the study of oxidative stress injury in cochlear spiral ganglion neuronal cells (SGNs), it was found that when GPX1 is knocked out, the oxidative stress damage of SGNs by peroxynitrite is aggravated, resulting in loss of SGNs and loss of hearing (Wang et al., 2022). All in all, the lack of GPX1 aggravates the damage caused by oxidative stress to tissues and organs. On the contrary, increasing the expression of GPX1 will bring protective effects to the corresponding tissues and organs. In studies of brain injury models, overexpression of GPX1 can improve spatial learning ability after brain injury in young mice compared with wild-type mice, possibly due to overexpression of GPX1, which reduces oxidative damage in mice at an early stage (Tsuru-Aoyagi et al., 2009). Similarly, using a GPX mimic Ebselen alleviates cerebral ischemia perfusion injury in GPX1−/−deficient mice (Wong et al., 2008). Importantly, Ebselen mimicked the activity of all selenium-dependent mammal GPXs and affected the redox state. Therefore, its protective effect overlaps with GPX-1.

Interestingly, we found in our study that GPX1 is not capable of oxidative stress in all cases. We mentioned that H_2_O_2_ acts as an important signaling molecule in oxidative stress damage, mediating the oxidative stress response of tissues and organs. However, in studies of acute lung injury caused by LPS, H_2_O_2_ mediates not only inflammatory damage, but also has anti-inflammatory effects on neutrophils (Zmijewski et al., 2009). LPS-mediated NFκB activation and pro-inflammatory cytokine production decreased in neutrophil NFκB led to a weakened inflammatory response in the acute phase of mice with acute lung injury, suggesting that H_2_O_2_ can inhibit the acute excessive inflammatory response (Lubos et al., 2010). Surprisingly, this result was consistent with the results of GPX1 KO mice compared to WT mice. In GPX1 KO mouse bronchoalveolar lavage fluid, LPS-mediator levels and macrophage numbers were lower. At this point, GPX1 surprisingly appears to enhance the initial inflammatory response by initiating the production of pro-inflammatory cytokines to enhance the LPS-induced inflammatory response (Bozinovski et al., 2012). At the same time, GPX1 KO mouse endothelial cells show higher adhesion molecule expression in response to LPS, while GPX-1 high expression mouse endothelial cells have decreased adhesion molecule expression (Lubos et al., 2010). This is the opposite of the traditional antioxidant stress effect of GPX1. No reasonable explanation has been found for these studies with opposite results. It may be related to the dose of the toxic drug used or the period of study observation.However, most of the current research results support the antioxidant stress effect of GPX1, so GPX1 is an important antioxidant in mammals.

### 2.2 GPX2

Glutathione peroxidase 2 (GPX2) is mainly expressed in the gastrointestinal system, including the intestinal epithelium, esophageal epithelial cells, etc., and can also be detected in the human liver (Esworthy et al., 2022). Therefore, it is also named GI-GPX or GPX-GI. GPX2 protein concentrations are highest at the bottom of the crypt and then gradually descend to the top of the colonic crypt or small intestinal villi (Florian et al., 2001). Since the base of the crypt is not the preferred site for absorption, blocking H_2_O_2_ uptake is difficult to make the main function of GPX2. Due to the peculiarities of the expression location of GPX2, it is considered the first barrier for mammals to prevent the absorption of hydroperoxides produced by food.Studies have found that the expression level of selenoprotein in mammals is not balanced with the supply of selenium. When selenium is reduced, some selenoproteins in the body quickly disappear (low grades), while others can continue to be synthesized even when selenium levels are reduced (high grades) (Banning et al., 2008b). The RNA of low-grade selenoproteins degrades rapidly during selenium deficiency, while the RNA of high-grade selenoprotein remains stable, resulting in increased selenium reuse and faster resynthesis of selenoproteins (Banning et al., 2008b).In the GPX family with Sec as the active center, GPX2 has the highest rating, followed by GPX4, GPX3 and GPX1 (Banning et al., 2008b). This result was confirmed in a study on the mouse colon, where only GPX1 and GPX3 RNA decreased, while the RNA of GPX2 and GPX4 remained stable when mice were deficient in selenium (Kipp, 2017). Therefore, based on the ranking in the hierarchy can be inferred that the functions of GPX2 may be more important than those of other GPXs.

**FIGURE 2:**
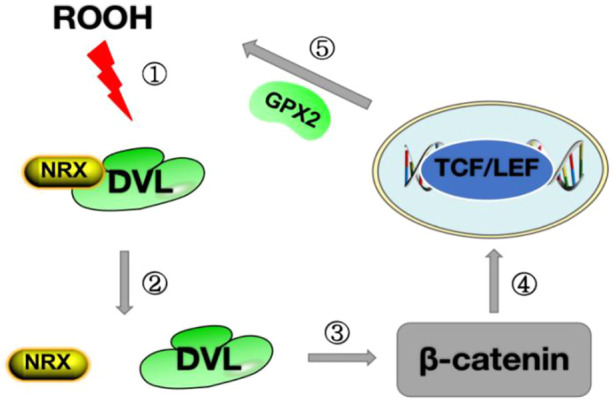
Diagram of the Wnt pathway mechanism.Step ①: ROOH oxidizes NRX on DVL;Step ②: The structure of NRX is changed after oxidation, releasing DVL;Step ③ After the release of DVL, inhibit *β*-catenin degradation and stabilize the expression of *β*-catenin in the cytoplasm; Step ④: *β*-catenin enters the nucleus and binds to TCF/LEF to promote the synthesis of GPX2;Step ⑤: GPX2 is released, ROOH is cleared, and oxidative stress is reduced.ROOH stands for lipid peroxide; DVL stands for Disheveled protein;NRX stands for Nuclear Redox Protein; GPX2 stands for glutathione peroxidase 2.

GPX2 was the first selenoprotein identified as a target of the Wnt pathway, and its promoter contains five β-catenin/TCF binding sites (TBE) (Li et al., 2020). The Wnt pathway controls the expression of genes required by stem cells to proliferate in the crypt floor region, which is the basic process for maintaining mucosal homeostasis. Colocalization of the Wnt pathway and GPX2, as well as the regulation of GPX2 through Wnt signaling, suggest that GPX2 plays a role in the continuous self-renewal of the intestinal epithelium and thus in the homeostasis of the intestinal mucosa (Pérez et al., 2017). We mentioned in our previous description that thiol oxidation is a common denominator in transcriptional regulation in the GPX family. Inflammatory cells transmit signals by releasing oxidative substances to stimulate the production of ROS. In the Wnt pathway, the key step of the Wnt pathway is the binding of the Wnt protein to the Frizzled receptor, and the nuclear redox protein (NRX) blocks Disheveled (DVL) and plays a negative regulatory role in the Wnt pathway (Yu et al., 2021). When NRX is oxidized by NADPH oxidase 1 (NOX1)-derived H2O2 or lipid peroxide (ROOH), DVL is released and inhibits glycogen synthesis kinase 3 (GSK3) in the β-catenin degradation complex, thereby stabilizing the β-catenin in the cytoplasm and partially β entering the nucleus to activate the target gene GPX2 by correlation with the TCF/LEF transcription factor family (Kipp et al., 2012) (Figure 2). The upregulation of GPX2 leads to the clearance of H_2_O_2_, the inhibition of apoptosis, and the weakening of oxidative stress. By removing hydroperoxides, GPX2 enhances the activity of the WNT pathway in the feedback loop. These effects of GPX2 have been demonstrated in oxidative stress injury studies of metastasis and apoptosis in cervical cancer (Wang et al., 2019).

The effects of GPX2 on oxidative stress are currently widely demonstrated.In the inflammatory response of bifidobacteria attenuating intestinal epithelial cells, upregulation of GPX2 can reduce oxidative stress and reduce the release of inflammatory factors (Ehrlich et al., 2020). At the same time, it has also been shown that GPX2 alleviates the apoptosis response of MCF7 cells to oxidative stress (Yan and Chen, 2006).Therefore, GPX2 is believed to have the effect of oxidative stress and reducing the inflammatory response.The inhibitory effect of GPX2 on oxidative stress is a key link in inhibiting tumor development.Therefore, the antioxidant function of GPX2 may provide an early defense against the development of colon cancer.In studies on human colon cancer cells, GPX2 prevents inflammatory factor-driven carcinogenic processes by inhibiting the expression of COX-2 and mPGES-1 in human colon cancer cells and reducing inappropriate responses to inflammatory stimuli (Banning et al., 2008a).The anti-inflammatory effect of GPX2 is confirmed in a study of the induction of inflammation-associated colon cancer in WT and GPX2 KO mice under AOM/DSS combined with different selenium content (Brigelius-Flohé and Kipp, 2012).The inflammatory response of GPX2 KO mice was generally heavier, especially in the moderate selenium deficiency group.In adequate selenium content, inflammation and tumor cell production are reduced (Brigelius-Flohé and Kipp, 2012).However, it is interesting to note that the tumor volume in GPX2 KO mice is smaller compared to WT mice, which proves that GPX2 supports the growth of tumor cells, which has been confirmed in studies in which GPX2 inhibits the metastasis and invasion of HT-29 adenocarcinoma cells (Banning et al., 2008b).This may be related to the anti-apoptosis effect of GPX2, and inhibition of apoptosis may also explain why tumors in WT cells grow better than those in GPX2 KO cells (Brigelius-Flohé and Kipp, 2012).In conclusion, GPX2 is a protective enzyme with oxidative stress and reduces inflammatory response, and its prominent role in inflammation seems to be prevention.Thus, GPX2 can stop tumorigenesis but support the growth of tumors that have already occurred.Therefore, the gain or loss of increased GPX2 expression mainly depends on the current state of the tumor and the stage of the tumor.

### 2.3 GPX3

Glutathione peroxidase 3 (GPX3) is a tetramer, containing two of the four arginines bound to GSH, Arg103 and 185 (Aumann et al., 1997).Extracellular GPX3, also known as GSHPX, is a selenoprotein with antioxidant potential. In all reported GPX families, the tetramer GPX3 eliminates all complex hydroperoxides, circulates in plasma in addition to its roles in cytoplasmic and mitochondria, and is a major ROS scavenger in plasma (Nirgude and Choudhary, 2021). The level of GPX3 can be measured using plasma, making it a useful prognostic and diagnostic biomarker. GPX3 mRNA is present in renal proximal tubular epithelial cells, and GPX3 may have specific antioxidant functions in the renal tubules or extracellular spaces due to the higher concentration of GSH in the kidneys. Approximately 70 percent of GPX3 is secreted by the basolateral membrane of renal proximal convoluted tubule (PCT) cells and into plasma through the basilar artery (Zhao et al., 2019; Nirgude and Choudhary, 2021). GPX3 is present not only on the surface of plasma and mucous membranes, protecting epithelial cells from oxidative damage. A major extracellular enzyme, GPX3, is also found in the cytoplasm and plasma membranes of the mammalian kidney, epididymis, heart, lung, liver, brain, adipose tissue, mammary gland, and gastrointestinal tract (Nirgude and Choudhary, 2021). Thus, kidney-derived GPX3 can be transported to other tissues and bound to the basement membrane. A special case is an epididymal tissue, which synthesizes GPX3 and releases it into a cavity, where sperm storage takes place (Burk et al., 2011). The lack of selenium in the body can significantly reduce the expression of GPX3. Therefore, GPX3 is believed to play a vital role in oxidative stress and reducing the inflammatory response.

GPX3 is not characteristic in transcriptional regulation, and the upregulation of GPX3 expression under inflammatory conditions is thought to be due to oxidative stress. GPX3, similar to GPX1, is located at a low level of the selenoprotein level and is sensitive to selenium content in the body, so it is often used as a biological marker to measure selenium content in the body. GPX3 can use glutathione as an excellent substrate to participate in the redox reactions involved in glutathione and NADPH (Figure 1). GPX3 is a typical N-terminal pilot sequence, but lacks endoplasmic reticulum (ER) preserved signal. This suggests that GPX3 is not reserved across the ER. In recent years, the role of GPX3 in oxidative stress has been reported, for example: GPX3 can inhibit the expression of HIF1-α by regulating ROS, thereby inhibiting the growth of melanoma cells (Yi et al., 2019). In the peroxisome proliferator-activated receptor γ (PPARγ)-induced COPD model, upregulation of GPX3 expression can reduce lung tissue damage caused by oxidative stress (Reddy et al., 2018). In addition to an increase in inflammation levels, the number of tumor cells also increased in the number of tumor cells after GPX3 KO during the development of colitis-related cancers (Barrett et al., 2013). This may be related to GPX3’s inhibition of oxidative stress and apoptosis levels.

**FIGURE 3:**
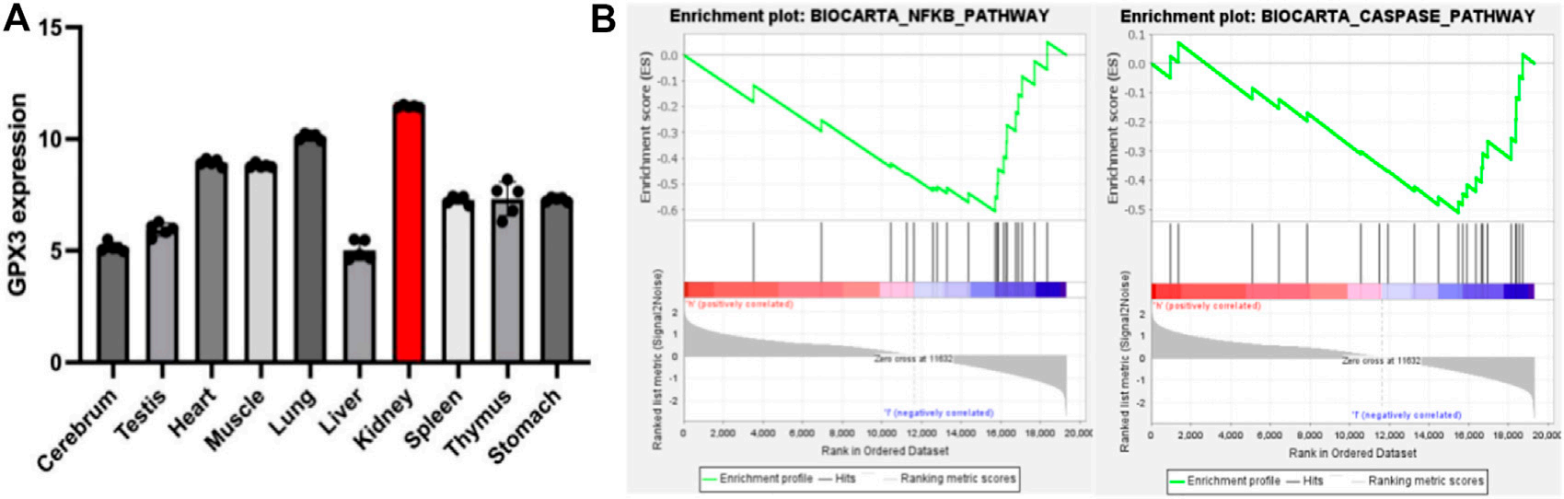
**A)** represents the statistical chart of the expression level of GPX3 in various tissues and organs under the physiological conditions of mice; **(B)** represents the enrichment of GPX3 pathways (NFκB pathway and Caspase pathway) after single-gene GSEA analysis in the renal ischemia-reperfusion injury model.

As mentioned earlier, the vast majority of GPX3 is secreted by the renal proximal convoluted tubules, so we speculate that the kidney may be a highly expressed organ of GPX3, and to test our hypothesis, we analyzed the GSE111159 dataset (This dataset mainly detects the gene sequencing of 10 tissues and organs of mouse kidney, liver, brain, lung, thymus, muscle, spleen, stomach, heart and testicle under physiological conditions to understand the gene expression of each tissue and organ) in the GEO database (Gene Expression Omnibus, http://www.ncbi.nlm.nih.gov/geo). We found that GPX3 had the highest level of expression in kidney tissue under mouse physiology (Figure 3A), which is consistent with the results of the analysis by Kim et al. (Kim et al., 2018). At the same time, we also performed single-gene GSEA analysis of GPX3 in the GSE148420 dataset (dataset of gene expression differences before and after renal ischemia-reperfusion). We found that GPX3 inhibits oxidative stress-related pathways (NFκB pathway) and apoptosis-related pathways (Caspase pathway) (Figure 3B), which is consistent with our previous results. At the same time, in our previous study on the protective effect of Ebselen on renal ischemia-reperfusion injury, we found that the expression level of GPX3 in renal tissues decreased after renal IRI, and when the level of GPX3 increased after the use of Ebselen, the pathological damage of renal histologic damage was alleviated, and we speculated that GPX3 may play an important role in the oxidative stress response of renal ischemia-reperfusion injury (Wu et al., 2022). In cisplatin-induced renal injury, MicroRNA-483-5p can induce the expression of GPX3 to weaken the oxidative stress response, thereby playing a protective role in renal injury (Xia et al., 2022). However, the relevant mechanism of GPX3 kidney injury is still unclear. Therefore, combined with the antioxidant stress effect of GPX3 and its high expression in kidney tissue, it may provide new therapeutic targets for studying kidney damage in the future.

### 2.4 GPX4

Glutathione peroxidase 4 (GPX4) is a membrane-associated phospholipid hydroperoxidase and the only enzyme in the GPX family that directly reduces and destroys lipid hydroperoxides, GPX4 is characterized early and characterizes as the lipid peroxidation inhibitory protein PIP, mainly due to its unique ability (Jia et al., 2020). In addition to H_2_O_2_ and small molecule hydroperoxides, it is also capable of reducing complex lipid compounds such as phospholipids, cholesterol and cholesterol lipid hydroperoxides. It is recognized as the key to destroying fatty acid hydroperoxides (Jia et al., 2020). GPX4 exists in the form of a monomer, and its activity is better than that of GPX1 when the selenium content in the body is low (Banning et al., 2008b). GPX4 has been found to have three different forms of isoforms, namely mitochondrial type (MGPX4), cytoplasmic form (CGPX4), and sperm karyotype (SNGPX4) (Bersuker et al., 2019). The GPX4 gene consists of 7 exons and 6 introns. In studies of different animal models, there are also significant differences in the distribution of GPX4. CGPX4 is ubiquitous in cells, while MGPX4 and SNGPX4 are mainly expressed in the testis and only a small amount in other tissues (Bersuker et al., 2019). The amount of GPX4 protein in tissues does not accurately reflect its activity distribution. This may reflect specific Se-dependent cellular functions, or differences in the levels of factors that activate GPX4. The activation-inactivation mechanism of GPX4 is currently unknown, but high activity evidence on differentiated spermatogenic cell membranes suggests that there may be some link between cell differentiation and peroxide levels.

**FIGURE 4:**
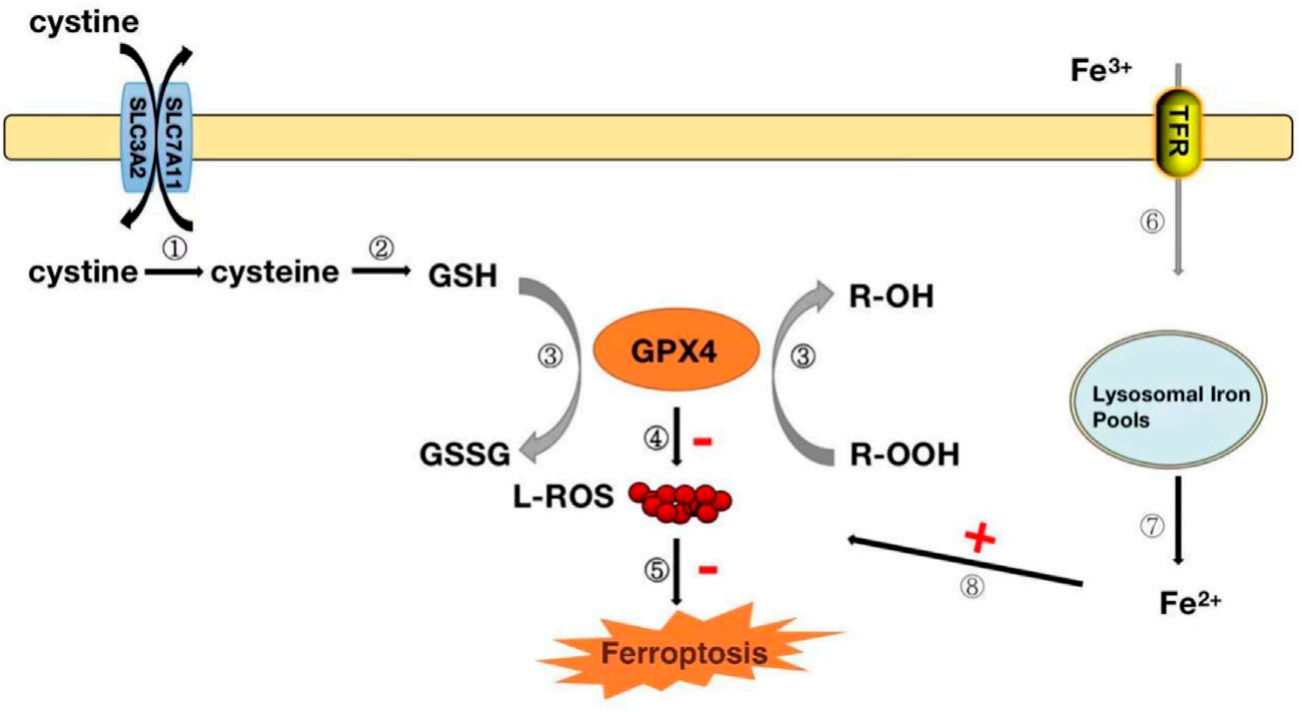
GPX4 modulates the mechanism of iron death.Steps ①-②: After the cystine outside the cell enters the cell, it is rapidly converted to cysteine for the synthesis of glutathione (GSH). Step ③: GPX4 converts reduced glutathione (GSH) to oxidized glutathione (GSSG) while reducing toxic lipid peroxide (R-OOH) to non-toxic alcohol (R-OH). Steps ④-⑤: Inhibit the production of reactive oxygen species and organize the occurrence of ferrozoticosis, on the contrary, when the activity of GPX4 is reduced, it will promote the formation of ROS and induce the occurrence of ferrozoosis; Steps ⑥-⑧: The circulating Fe^3+^ enters the cell *via* transferrin and is reduced to Fe^2+^, which when Fe^2+^ is excessive, can lead to the accumulation of ROS and induce the occurrence of ferrozoticosis.

Ferrozois, a new form of regulation of cell death, is primarily attributed to cellular lipid peroxidation (LPO) due to insufficient or absent GPX4 activity, ultimately causing cell death (Forcina and Dixon, 2019). There appears to be some close association between the antioxidant effects of GPX4 and ferrozois. Iron death has also become one of the hot issues in the current research on the development of diseases. The interaction between ferrozois and GPX4 has been elaborated in detail, and we will only give a brief overview here. GPX4 converts reduced glutathione (GSH) to oxidized glutathione (GSSG) and reduces toxic lipid peroxides (R-OOH) to corresponding alcohols (R-OH) or free hydrogen peroxide (H_2_O_2_) to water (Ingold et al., 2018; Forcina and Dixon, 2019; Ursini and Maiorino, 2020). The Xc-System is an important antioxidant system consisting of glycosylated heavy chain SLC3A2 and non-glycosylated SLC7A11. With their assistance, cystine is absorbed into cells and rapidly reduced to cysteine, participating in the synthesis of intracellular GSH (Ingold et al., 2018; Ursini and Maiorino, 2020) (Figure 4). Inhibition of the Xc-system determines the decrease in GSH levels, which indirectly leads to the inactivation of GPX4 and the onset of ferroptosis (Ingold et al., 2018; Ursini and Maiorino, 2020). Normal iron is essential for the survival of living organisms and is an essential element in many biological processes. Circulating Fe^3+^ enters cells with the assistance of transferrin and subsequently converts to Fe^2+^ (Ingold et al., 2018). However, when intracellular iron ions are overloaded, Fe^2+^ can catalyze the generation of metabolically toxic reactive oxygen species and phospholipid peroxide through the Fenton reaction, leading to cell damage or death (Ingold et al., 2018) (Figure 4). Therefore, the uptake, release, storage and transport of iron ions in cells are all important factors affecting iron death. Inhibition of the Xc-system, inactivation of GPX4 and overloading of iron ions are the three most critical factors leading to ferroptosis. These factors have been confirmed in studies. In the study of glioblastoma, RSL3 inhibits the activity of GPX4, drives the occurrence of ferrozois and induces oxidative death of glioblastoma cells, providing new therapeutic ideas for glioblastoma (Li et al., 2021). Prominin2 is a protein related to the regulation of lipid dynamics, which can promote the formation of ferritin-containing multivesicular bodies (MVB) and exosomes, transport iron out of cells, and inhibit ferroptosis. Play an important role in the ferroptosis resistance of cancer cells (Brown et al., 2019). In studies of myocardial ischemia-reperfusion injury, naringenin can provide a protective effect on oxidative stress damage caused by myocardial ischemia-reperfusion by modulating the Xc-system and inhibiting iron death (Xu et al., 2021).

In addition to the iron death we mentioned above, GPX4 also plays an important role in the process of sexual maturation in males. Spermatozoa have the highest selenium content in mammalian tissues, and the need for selenium increases at the onset of spermatogenesis (Wang et al., 2021). An inadequate selenium supply impairs the synthesis of the mitochondrial envelope of sperm, which affects sperm motility and may lead to infertility (Wang et al., 2021). Selenium supplementation in infertile men has been found to increase selenium concentrations in semen and improve sperm motility (Su et al., 2021). Nevertheless, SNGPX4 KO mice have normal survival and fertility. Morphologically, no significant abnormalities were seen in the testes and spermatozoa of mice after SNGPX4 KO (Gharagozloo et al., 2016). However, sperm from the head and tail of the epididymis in SNGPX4 KO mice are more prone to decline than WT mice at various stages of epididymis maturation, suggesting that SNGPX4 plays an important role in stabilizing the structural integrity of sperm chromatin (Gharagozloo et al., 2016). In short, as an important antioxidant enzyme in mammals, GPX4’s role in the oxidative stress response cannot be ignored.

### 2.5 GPX5

Glutathione peroxidase 5 (GPX5), an epididymis-specifically expressed selenium-free antioxidant enzyme, was first isolated and identified in the epididymal tissues of mice (Taylor et al., 2013). GPX5 is an unusual member of the mammalian GPX family because, unlike the well-known mammalian GPX1-4, it does not contain selenosylcysteine (Secys) at its active site, but instead has cysteine residues (Cys) as its active site (Taylor et al., 2013). The epididymis is where sperm matures, transports, and stores. ROS is a product of cellular redox metabolism, and moderate amounts of ROS in the epididymis can play a role in sperm maturation and capacitation by regulating cAMP levels (Luan et al., 2019). Spermatozoa are particularly susceptible to increased ROS levels because their membranes contain more polyunsaturated fatty acids, which are extremely sensitive to peroxide reactions. Since sperm contain only a small amount of cytoplasmic antioxidants, most of the cytoplasm has been shed. Therefore, they rely on protecting antioxidant substances synthesized and secreted by epididymal epithelial cells, including glutathione peroxidase 5 (GPX5) (Taylor et al., 2013). Therefore, the balance between ROS production and antioxidant substances in the epididymis is essential for sperm development and maturation. In studies of oxidative stress in epididymal epididymal cells in goats, miR-542-3p mRNA can downregulate the expression of GPX5, thereby aggravating oxidative damage in epididymal epididymal cells (Yang et al., 2022). In the study of the protective effect of trehalose on oxidative stress damage in sheep epididymal epididymal cells, it was again found that the upregulation of GPX5 plays a key role (Luan et al., 2021). It can be seen that GPX5 is essential in regulating oxidative stress damage in the epididymis and protecting sperm maturation, which is of great significance in maintaining the reproductive ability of mammals.

During epididymis transport, spermatozoa are vulnerable to intracellular and extracellular ROS attacks and rely on antioxidant scavengers synthesized by the epididymis for protection. GPX5 plays an important role as part of this protection system. GPX5 is synthesized primarily by epididymal head epithelial cells and secreted into the epididymal cavity to compensate for a deficiency of antioxidant enzymes in immature sperm (Lahti et al., 2001). The concentration of GSH in the head is significantly higher than in the tail, and there is a clear parallel relationship between the GSH concentration and the content of GPX5, which makes the regulation of oxidants by GPX5 possible (Seligman et al., 2005). In studies of GPX5 knockout mice, we deduced that GPX5 is a way to maintain the integrity of sperm DNA function by removing excess H_2_O_2_, and these mice did not exhibit significant defects in the epididymis and sperm cells (Chabory et al., 2009). In addition, in GPX5 KO male mice, fertility is not affected at a young age. However, mating WT female mice with 1-year-old GPX5 KO male mice found a higher incidence of offspring miscarriage and developmental malformations, which are related to oxidative stress damage in sperm (Chabory et al., 2009). In the study of SNGPX4 and GPX5 dual knockout (DKO) mice, when the double gene knockout occurred, the mice showed abnormalities in the sperm nucleus structure. An increase in the activity of H_2_O_2_ scavengers was detected (Noblanc et al., 2012), proving that after SNGPX4 and GPX5 double knockouts, the epididymal tissues of mice produced oxidative stress responses, indicating that SNGPX4 and GPX5 play a role in maintaining the normal structure of the sperm nucleus in the epididymal tissue (Noblanc et al., 2012). Similarly, resistance to oxidative attack increased after overexpression of GPX5 in CHO-K1 cells, and GPX5 overexpressing cells reduced lipid peroxidation levels and downstream DNA damage marker 8-OXODG levels compared to control cells (Taylor et al., 2013). In GPX5-gene KO mice, sperm DNA damage is significantly increased, while an 8-weeks pretreatment of GPX5 KO mice using antioxidants completely protects sperm DNA from oxidative stress damage (Gharagozloo et al., 2016). In summary, GPX5, as an antioxidant enzyme highly expressed in epididymal tissues, plays an important role in maintaining the microenvironment of the epididymis, protecting sperm from oxidative stress, and maintaining the integrity of DNA structure.

### 2.6 GPX6

Glutathione peroxidase 6 (GPX6), also known as olfactory organ glutathione peroxidase (Brigelius-Flohé and Maiorino, 2013), is a closely homologous protein with GPX3. Compared with other GPX family members, the expression of GPX6 is specific, and in the human body it is a selenoprotein with selenocysteine (Secys) as the active center (Brigelius-Flohé and Maiorino, 2013); However, in other rodents or mammals, selenocysteine cannot be synthesized due to the lack of selenocysteine insertion sequences in related genes, and it is a non-selenium protein replaced by cysteine (Cys) (Brigelius-Flohé and Maiorino, 2013). Similar to GPX3, it is also a protein expressed in tetramer form.

It is believed that GPX6 is mainly expressed in embryonic and olfactory organ epithelial cells, and may be involved in the transmission and degradation of odor-related signals. However, there are no relevant studies to confirm it. So far, GPX6 has not been purified and kinetic analysis results are unavailable, resulting in minimal understanding of GPX6. However, in a recent study on the inhibition of Hepa1-6 cell proliferation induced by oxidative stress, we found that increased expression of GPX6 protein may play a role in inhibiting oxidative stress (Tan et al., 2022). Furthermore, in the study of the neuroprotective effect of mesenchymal stem cells on the regulation of oxidative stress on retinal nerves, it was also found that the expression of GPX6 was upregulated (Usategui-Martín et al., 2022). The upregulation of GPX6 expression found in these two studies suggests that its upregulation may have some antioxidant effects. However, there are no relevant research reports on the mechanism of action of GPX6, which may be related to the oxidative stress response involved in glutathione. Further studies may be needed to reveal the function and mechanism of GPX6.

### 2.7 GPX7

Glutathione peroxidase 7 (GPX7) is a non-selenocysteine active neutral antioxidant enzyme in mammals, also known as non-selenium cysteine phGPX (NPGPX) due to its homology to phospholipid peroxide GPX (phGPX = GPX4) (Chen et al., 2016). However, unlike GPX4, GPX7 does not contain a GSH-binding domain, and when expressed and purified in *E. coli*, there is little GPX activity (Lénon et al., 2020). The mammalian GPX7 gene contains 3 exons, encodes 187 amino acids, and has a predicted molecular weight of 22 kDa. GPX7 has two unique endoplasmic reticulum secreted proteins, the N-terminal endoplasmic reticulum signaling peptides (HGPX7 and MGPX7) and the C-terminal atypical KDEL sequence (endoplasmic reticulum recovery signaling) (Raykhel et al., 2007). Although most GPX7 is found in the endoplasmic reticulum, it also appears to be expressed in other cells. When N-terminal signaling is cut, GPX7 can be transferred from the endoplasmic reticulum to the Golgi apparatus along the secretory pathway (Yoboue et al., 2017). As a non-selenium cysteine-containing GPX, GPX7 encodes cysteine (Cys57 and Cys86) at its catalytic site. Under the ROS stimulation, the thin-sophisticated moiety (-SH) of cysteine residues is oxidized to form reversible intramolecular disulfide bonds (R-S-S-R) and sulfinyl groups (R-SOH), or irreversible sulfinic acids (R-SO_2_H) and sulfonic acids (R-SO_3_H) (Kanemura et al., 2020). In studies of non-alcoholic steatohepatitis due to oxidative stress, overexpression of GPX7 in LX-2 cells will result in inhibition of ROS production and a decrease in the expression of profibrotic and pro-inflammatory genes (Kim et al., 2020). However, knocking down GPX7 in LX-2 cells increases the synthesis of profibrotic and pro-inflammatory genes and collagen (Kim et al., 2020). It can be seen that GPX7 plays an important role in oxidative stress and reducing the inflammatory responses in tissues and organs.

**FIGURE 5:**
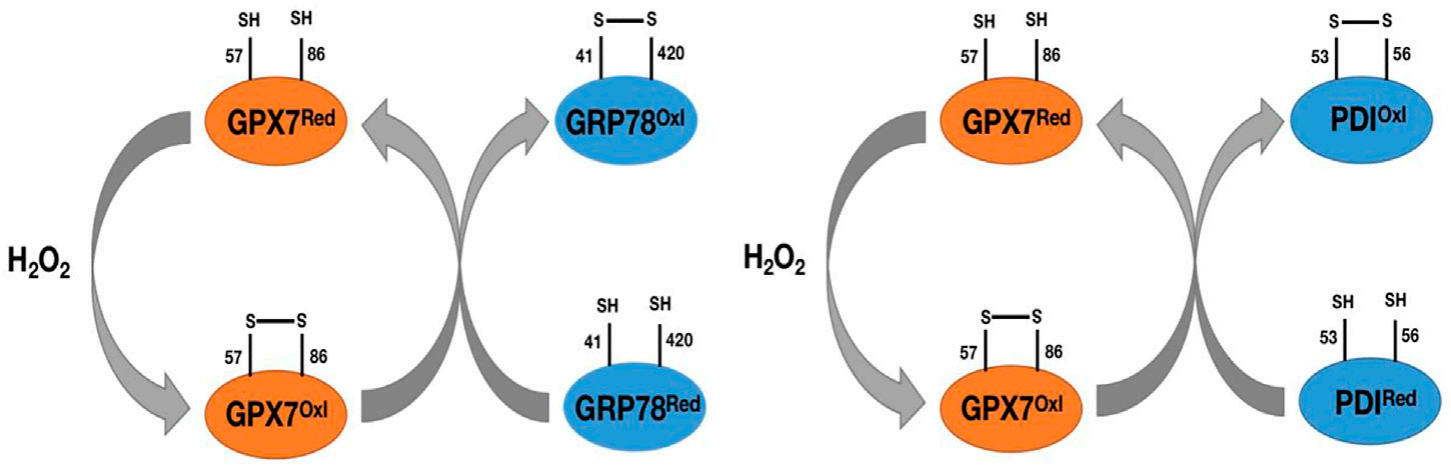
Pat plot of the chaperone proteins GRP78 and PDI versus GPX7. H2O2 catalyzes the formation of disulfide bonds between Cys57 and Cys86 on GPX7, prompts the transformation of GPX7 from reduced to oxidized form, and then oxidized GPX7 promotes the formation of disulfide bonds between Cys41 and Cys420 (Cys53 and Cys56) on GRP78 (PDI) and enhances the activity of GRP78(PDI) protein.

As we mentioned earlier, GPX7 does not contain domains bound to GSH, so it cannot participate in redox reactions with GSH as a substrate like other GPX family members. However, in response to oxidative stress, GPX7 can promote stress signal transduction and release through interaction with corresponding targeted proteins. The most important are the 78 kDa glucose regulatory protein (GRP78) and protein disulfide isomerase (PDI). GRP78, also known as immunoglobulin heavy chain binding protein (BIP), is one of the main chaperone proteins in the endoplasmic reticulum (ER) (Wei et al., 2012). ER is a center for the biosynthesis of extracellular and organelle proteins. Under unfolded protein stress conditions, GRP78 binds to unfolded or misfolded proteins and subsequently activates downstream ER stress sensors, including IRE1, PERK, and ATF6, to trigger a response to unfolded proteins (Yoboue et al., 2017)^,^ (Wei et al., 2012). The unfolded protein reaction will weaken weakened translation, upregulation of chaperone proteins, and degradation of misfolded proteins. In order to be successfully synthesized in ER, the chaperone GRP78 is induced and activated to promote the refolding process of misfolded proteins (Yoboue et al., 2017)^,^ (Wei et al., 2012). GPX7 can transmit oxidative stress signals by forming disulfide bonds between its catalytic sites Cys57 and Cys86 residues, and this oxidized form of GPX7 can bind to each other with GRP78 and form covalent bond intermediates between Cys86 of GPX7 and Cys41/Cys420 of GRP78 (Nguyen et al., 2011; Wei et al., 2012) (Figure 5). Subsequently, the formation of disulfide bonds between Cys41 and Cys420 of GRP78 enhances its chaperone protein activity (Nguyen et al., 2011; Wei et al., 2012) (Figure 5). Loss of GPX7 in animals leads to an enhancement of systemic oxidative stress (Wei et al., 2012). Therefore, GPX7 can maintain physiological homeostasis by enhancing the activity of GRP78 chaperone protein.

**FIGURE 6:**
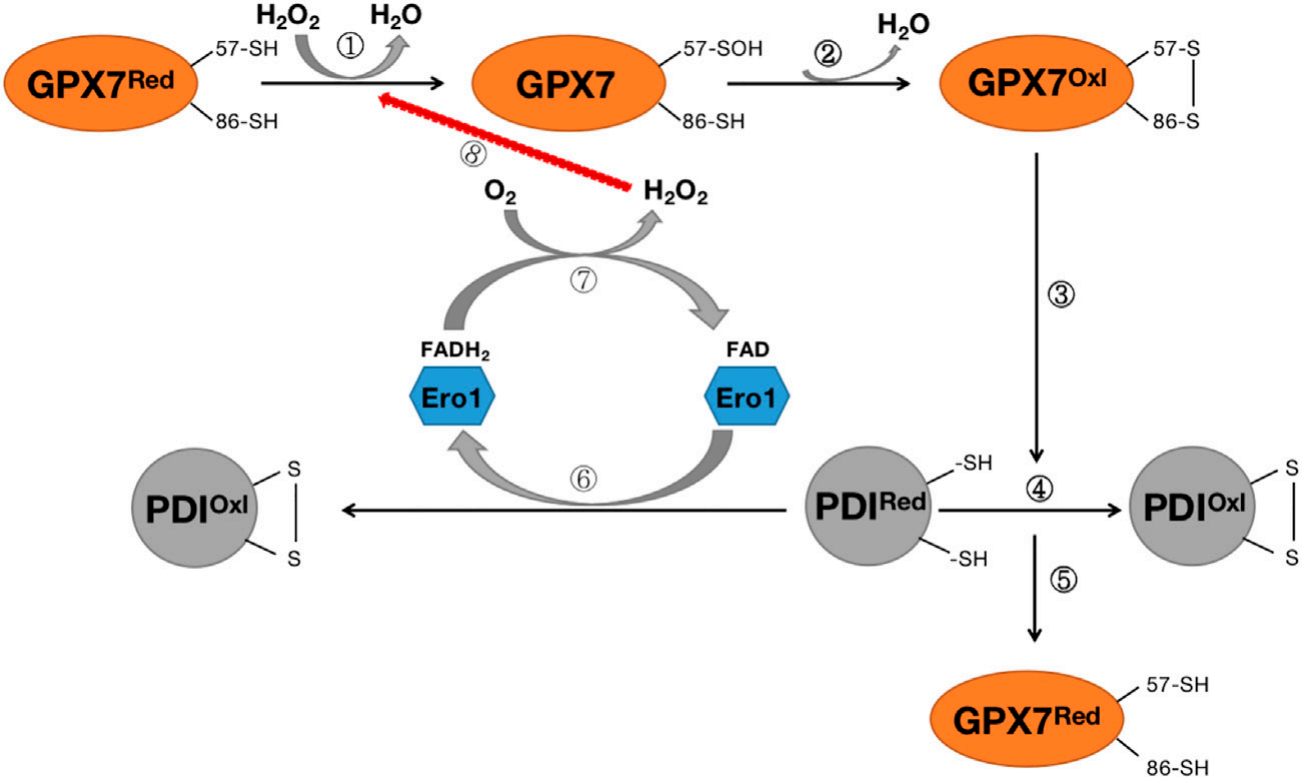
Diagram of the Rro1/GPX7/PDI protein folding mechanism. Steps ①-②: H_2_O_2_ converts reduced GPX7 into oxidized GPX7 and releases a subset of H_2_O; Steps ③-④: Oxidized GPX7 can promote the formation of disulfide bonds on PDI, convert reduced PDI into oxidized PDI, and enhance the activity of PDI; Step ⑤:P during the oxidation of DI, GPX7 is re-transformed from oxidized form to reduced form; Steps (6)–(8): The reduced PDI can be oxidized by endoplasmic reticulum oxidized protein 1 (Ero1) to convert to oxidized PDI, while transferring 2 electrons to O_2_, thereby forming H_2_O_2_, which again participates in the oxidation process of GPX7. GPX7^Red^ stands for reduced glutathione peroxidase 7; GPX7^Oxl^ stands for oxidized glutathione peroxidase 7; PDI^Red^ stands for reduced protein disulfide isomerase enzyme; PDI^Oxl^ stands for oxidized protein disulfide isomerase enzyme; ERO1 stands for endoplasmic reticulum redox protein 1.

The primary source of ROS in the endoplasmic reticulum is the formation of intramolecular disulfide bonds during the maturation of many secreted proteins and membrane proteins. This process requires the involvement of protein disulfide isomerase (PDI), ER redox protein 1 (ERO1), and GRP78 chaperone protein. The ERO1-PDI axis is thought to be the main pathway for disulfide bond formation in ER (Wang et al., 2014). ERO1 plays a vital role in the ER redox process and rapidly converts its activity to maintain a balanced redox environment, thereby facilitating the smooth process of protein folding (Bosello-Travain et al., 2013; Wang et al., 2014). In an oxidative ER environment, activated ERO1 generates disulfide bonds by consuming O_2_ in the presence of flavin cofactors, which are then passed to the protein for folding *via* PDI (Bosello-Travain et al., 2013; Wang et al., 2014). GPX7 can utilize the H_2_O_2_ produced by Ero1 to accelerate the oxidative folding process of proteins *in vitro* and *in vivo* (Bosello-Travain et al., 2013; Wang et al., 2014) (Figure 6). GPx7 likes to interact with the domain of PDI, and intramolecular cooperation between the two redox-active sites of PDI increases the activity of the Ero1α/GPx7/PDI triplet, thereby promoting protein folding mechanisms (Bosello-Travain et al., 2013; Wang et al., 2014) (Figure 6). Although the knockout of GPX7 does not lead to the direct death of mice, the production of excess reactive oxygen species leads to incorrect oxidative modification of proteins and irreversible changes in function, resulting in various diseases.

In summary, GPX7 is an essential sensor for oxidative stress and endoplasmic reticulum stress (Chen et al., 2016). The redox form of GPX7 is determined by the redox state of the cell. When oxidative stress occurs in cells, GPX7 is activated and transfers disulfide bonds to specific proteins, increasing protein activity in response to oxidative stress. For example, it promotes protein folding in the ER by regulating the expression of chaperone proteins such as GRP78 and PDI. Therefore, the increase in the activity and quantity of GPX7 is critical for the body’s oxidative stress response. GPX7 still has a unique role in maintaining redox homeostasis despite being different from other GPX in response to oxidative stress.

### 2.8 GPX8

Glutathione peroxidase 8 (GPX8) is a type II transmembrane protein with rare structural features that consists of 209 amino acids (Yoboue et al., 2017). GPX8 is similar to GPX7 because both have low glutathione peroxidase (GSH) activity, mainly due to the lack of domains bound to GSH. At the same time, it has a similar function to GPX7, which can increase the PDI activity of ER redox protein 1 (ERO1), promote the oxidative folding of endoplasmic reticulum proteins, and reduce oxidative stress (Raykhel et al., 2007). However, unlike GPX7, which is a transmembrane protein that contains a C-terminal KEDL endoplasmic reticulum localization sequence and a highly conserved N-terminal transmembrane domain (TMD) (Yoboue et al., 2017). It has been proposed that overexpression of GPX8 reduces Ca^2+^ storage and histamine-induced Ca^2+^ release in the endoplasmic reticulum (Yoboue et al., 2017); In HeLa cells, GPX8 is enriched in mitochondria-associated membranes (MAMs), a critical integrating center for calcium, lipid metabolism, and redox signaling homeostasis. When GPX8 is overexpressed, it leads to a decrease in Ca^2+^ levels in the endoplasmic reticulum (Yoboue et al., 2017). Conversely, when GPX8 is silenced, the histamine-induced release of Ca^2+^ from the endoplasmic reticulum to mitochondria and cytoplasm is increased (Yoboue et al., 2017). The regulatory effect of GPX8 on Ca^2+^ may be related to the inositol 1,4,5-triphosphate receptor (IP3R) effect, and the transmembrane domain (TMD) of GPX8 is thought to play a key role in the regulation of Ca^2+^ signaling (Yoboue et al., 2017). Therefore, GPX8 not only participates in the folding of proteins in the endoplasmic reticulum, exerts the role of oxidative stress, but also participates in the regulation of Ca^2+^ in the endoplasmic reticulum.

**FIGURE 7:**
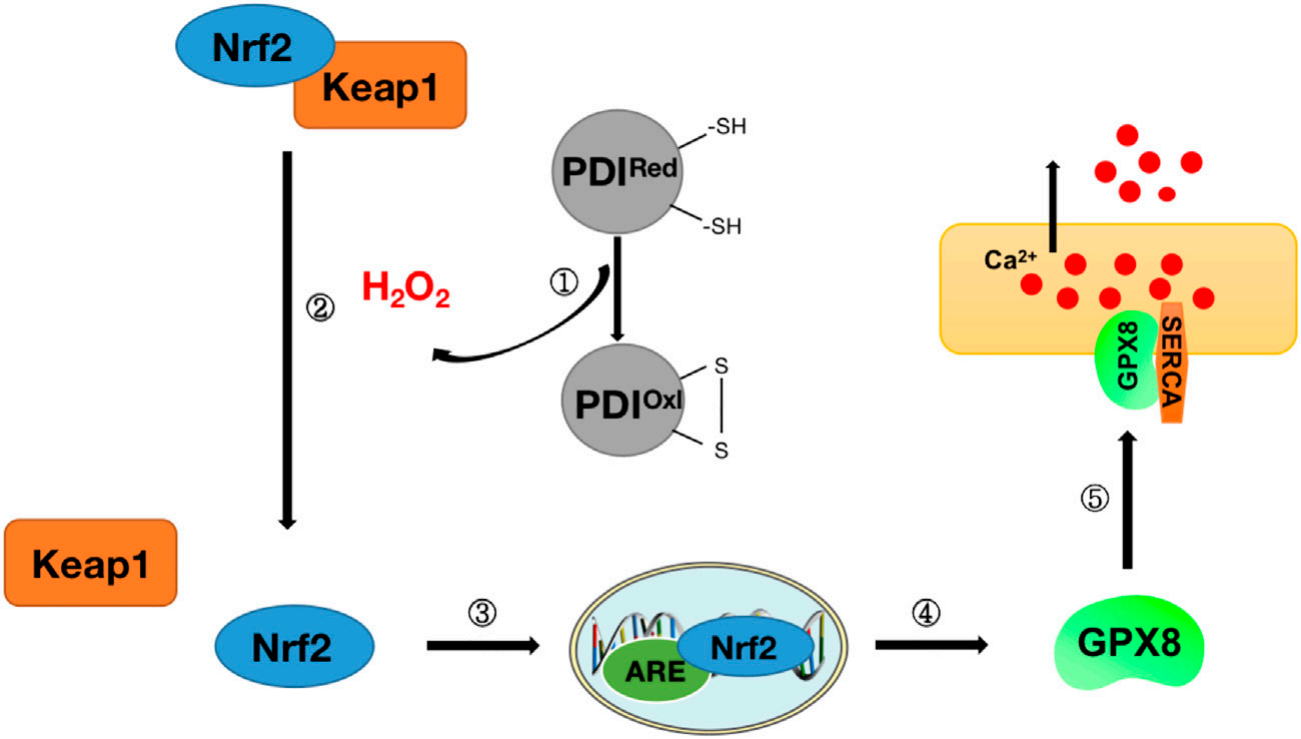
Diagram of protein oxidative folding-Nrf2-ER calcium axis mechanism. Step ①: Release of H_2_O_2_ during protein oxidative folding (see Figure 6 for details of protein oxidative folding); Step ②: Nrf2 and Keap1 complexes dissociate under H_2_O_2_ stimulation to release Nrf2; Steps ③-④: Nrf2 enters the nucleus and binds to antioxidant response elements (ARE) to stimulate the release of downstream antioxidant GPX8; Step ⑤: GPX8 interacts with SERCA to regulate Ca^2+^ in the endoplasmic reticulum.

Studies have found that there may be a link between the oxidative folding function of proteins in the endoplasmic reticulum and the regulatory function of Ca^2+^. H_2_O_2_ produced during the oxidative folding of endoplasmic reticulum proteins has been found to regulate the signal transduction process of nuclear factor E2-related factor 2 (Nrf2), which in turn regulates the expression of downstream glutathione peroxidase 8 (GPX8), which in turn can participate in ER calcium regulation through endoplasmic reticulum calcium ATPase (SERCA) (Granatiero et al., 2019) (Figure 7). These findings tightly link ROS and Nrf2 to ER oxidative protein folding and calcium signaling, establishing a regulatory mechanism for protein oxidative folding-Nrf2-ER calcium axis (Figure 7). This mechanism was also confirmed in subsequent studies. H_2_O_2_ signaling leads to the release of Nrf2 from complexes with Kelch-like ECH-associated protein 1 (Keap1) and ubiquitin ligase Cullin 3 and into the nucleus, where it binds to antioxidant response elements (ARE) and activates the expression of its target gene GPX8, which in turn regulates the expression of ER calcium by regulating SERCA (Granatiero et al., 2019). When HeLa cells are treated with Ero1 inhibitor (EN460), ER oxidative protein folding is impaired, reducing H_2_O_2_ signaling and inhibiting Nrf2 translocation to the nucleus, resulting in a decrease in GPX8, resulting in increased ER calcium content and signaling (Granatiero et al., 2019). To further confirm Nrf2’s involvement in the ER calcium regulation process *via* GPX8, in the same study, after silencing Nrf2 in HeLa cells, GPX8 mRNA and protein levels also decreased, resulting in elevated ER and cytosolic calcium levels at rest and increased histamine-induced ER calcium release (Granatiero et al., 2019). The importance of oxidized protein folding-Nrf2-ER calcium axis regulation mechanisms has also been found in cell culture models of familial amyotrophic lateral sclerosis (ALS) with superoxide dismutase (SOD1)-muted (Tokuda et al., 2019).

In summary, the antioxidant stress of GPX8 is not only related to the oxidative folding of endoplasmic reticulum proteins, but also may play a vital role in regulating endoplasmic reticulum Ca^2+^. This also provides new ideas for the treatment of oxidative stress. However, there are few studies on the function of GPX8, and further research is needed to clarify the function and mechanism of GPX8 in the future.

## 3 Conclusion

Reactive oxygen species (ROS) is an umbrella term that contains oxygen radicals and non-radical compounds such as hydrogen peroxide (H_2_O_2_), hydroxyl radicals, lipid radicals, and (phosphorus) lipid hydroperoxides (Liu et al., 2022). ROS has long been recognized as a harmful substance, and its excessive accumulation will cause the release of inflammatory factors, enhance the adhesion of inflammatory factors to endothelial cells, and promote apoptosis and even cause cell death (Forrester et al., 2018). The glutathione peroxidase (GPX) family is one of the main enzymes that protects cells from oxidative stress. In general, GPX exerts the role of oxidative stress by catalyzing the reduction of H_2_O_2_ and organic hydroperoxides to water and corresponding alcohols, respectively.

**FIGURE 8:**
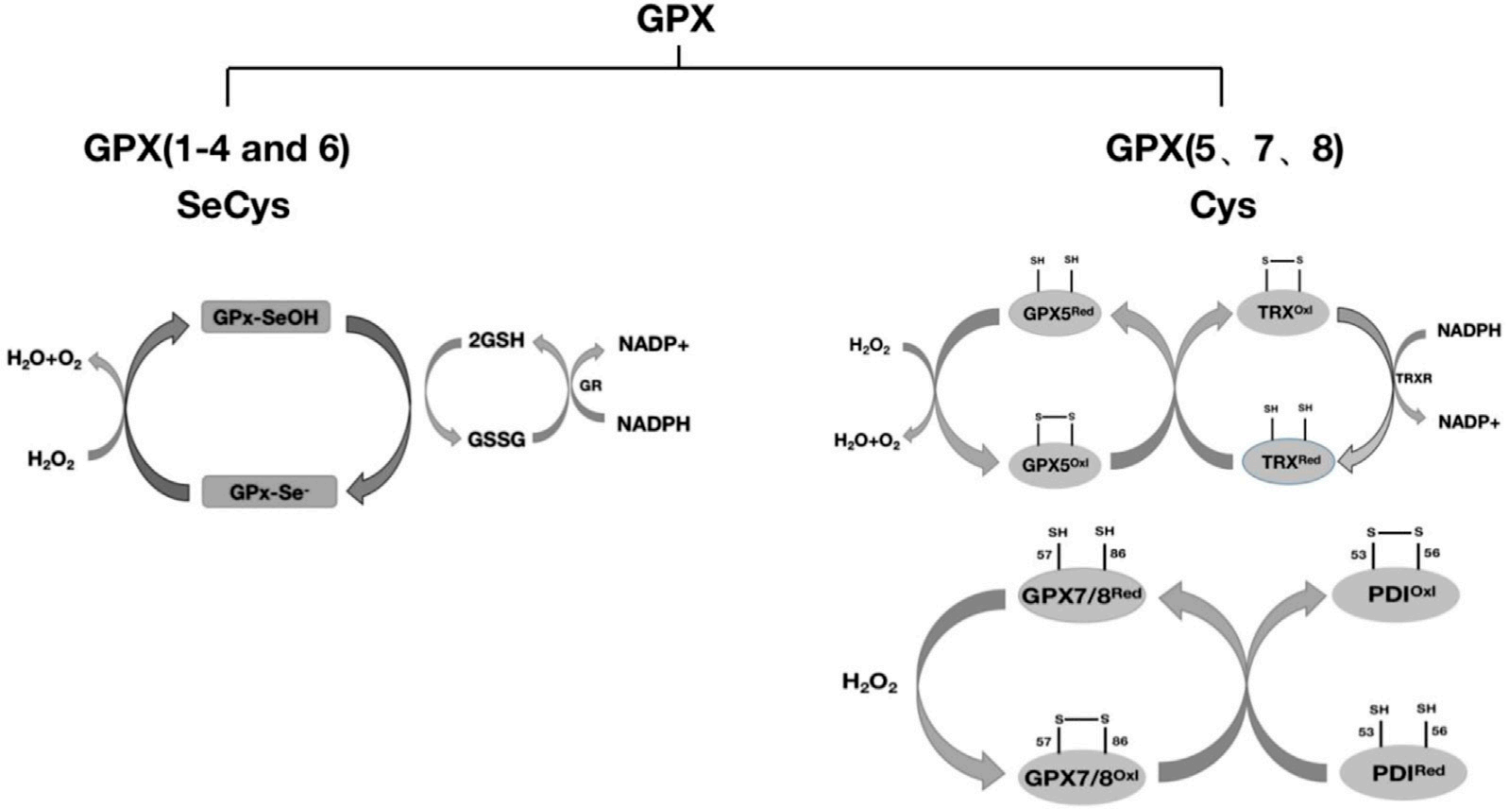
Classification of GPX and redox reactions. GPX1-4 and GPX6 reduce H_2_O_2_ with the assistance of glutathione (GSH), and whether GPX6 passes through this pathway remains to be further studied. GPX5 requires the reduction of H_2_O_2_ with the assistance of TRX. GPX7 and GPX8 require the reduction of H_2_O_2_ through the protein oxidative folding process with the participation of the chaperone protein PDI. The detailed process is described in this article.

The GPX family contains eight members (GPX1-GPX8), of which GPX1-GPX4 and GPX6 (human) members have an active center of selenocysteine, which is an important selenoprotein in mammals (Table 1); The active centers of GPX5, GPX7 and GPX8 are mainly cysteine. GPX1-4 and GPX6 are neutral to selenocysteine, so they can mediate redox reactions by interacting with GSH (Figures 1, 8). Among them, GPX4 is a membrane-associated phospholipid hydroperoxidase, and it is also the only enzyme in the GPX family that directly reduces and destroys lipid hydroperoxides, which can reduce complex lipid compounds in addition to reducing H_2_O_2_ and small molecule hydroperoxides; It is therefore recognized as a critical enzyme for destroying fatty acid hydroperoxides. In addition, GPX4 is also crucial in mediating the process of ferrozois, and the reduction of GPX4 activity has been found to promote the progression of ferrozoosis. GPX5 does not contain selenium cysteine (Secys) at its active site, but instead uses cysteine residues (Cys) as its active site. As an antioxidant enzyme with high expression in epididymal tissues, GPX5 plays an important role in maintaining the microenvironment of the epididymis, protecting sperm from oxidative stress, and maintaining the integrity of DNA structure. GPX7 and GPX8 also have cysteine as their active site and low glutathione peroxidase (GSH) activity, mainly due to the lack of domains bound to GSH. They were initially defined as secretory GPX, but later studies have shown that both enzymes are localized to the endoplasmic reticulum and are necessary enzymes involved in the oxidative folding of endoplasmic reticulum proteins (Figure 8). At the same time, GPX8 also plays a vital important role in endoplasmic reticulum Ca^2+^ regulation.

**TABLE 1:** GPX family structural characteristics.

GPX type	Revitalization center	Human expression (YES/NO)	Structure	Primary substrate	Expression sites
GPX1	Sec	YES	Tetramer	H_2_O_2_	Cytoplasm Mitochondria
				Hydroperoxides	
GPX2	Sec	YES	Tetramer	Hydroperoxides	Gastrointestinal tract
GPX3	Sec	YES	Tetramer	H_2_O_2_	Renal Plasma
				Hydroperoxides	
GPX4	Sec	YES	Monomer	H_2_O_2_	Testicles
				Hydroperoxides	
				Lipid peroxides	
GPX5	Cys	NO	Tetramer	H_2_O_2_	Epididymis
GPX6	Sec(Human)	YES Tetramer YES	H_2_O_2_ Tetramer	Olfactory organs H_2_O_2_	
	Cys(Rats and mice)				
GPX7	Cys	NO	Monomer	H_2_O_2_	Endoplasmic reticulum
GPX8	Cys	NO	Monomer	H_2_O_2_	Endoplasmic reticulum

## 4 Prospect

In-depth study of GPX family members has helped to discover not only the function of individual GPXs, but also the function of hydroperoxides as mediators in redox regulation. In addition to the role mentioned above in the process of oxidative stress, the GPX family also plays a unique role in tumor development (Laribi et al., 2021), viral infection (Strycharz-Dudziak et al., 2020), obesity (Gusti et al., 2021), apoptosis (Rusetskaya et al., 2019), signal transduction, and inflammation (Rusetskaya et al., 2019). However, how to give full play to the highly specific functions of GPX family members is currently a huge challenge. Due to differences in their distribution in tissues or cells and differences in known substrates, it is not known whether GPX family members can complement each other. At present, many studies have found that the supplementation of organic selenium compounds can improve the activity of GPX family members and enhance antioxidant effects (Mamgain et al., 2022). With the development of technology, the synthesis of different types of organic selenium compounds will greatly enhance the role of GPX family members. However, further research is needed to fully exploit the role of GPX. Therefore, the synthesis of new organoselenium compounds in the future provides new options for fully utilizing the functions of GPX family members. At the same time, targeting them in the future may lead to new treatment options for patients with associated diseases.

## References

[B1] Alehagen U. Opstad T. B. Alexander J. Larsson A. Aaseth J. (2021). Impact of selenium on biomarkers and clinical aspects related to ageing. A review. Biomolecules 11 (10), 1478. 10.3390/biom11101478 34680111 PMC8533247

[B2] Aumann K. D. Bedorf N. Brigelius-Flohé R. Schomburg D. Flohé L. (1997). Glutathione peroxidase revisited–simulation of the catalytic cycle by computer-assisted molecular modelling. Biomed. Environ. Sci. 10 (2-3), 136–155. 9315305

[B3] Banning A. Florian S. Deubel S. Thalmann S. Müller-Schmehl K. Jacobasch G. (2008). GPx2 counteracts PGE2 production by dampening COX-2 and mPGES-1 expression in human colon cancer cells. Antioxid. Redox Signal 10 (9), 1491–1500. 10.1089/ars.2008.2047 18479189

[B4] Banning A. Kipp A. Schmitmeier S. Löwinger M. Florian S. Krehl S. (2008). Glutathione peroxidase 2 inhibits cyclooxygenase-2-mediated migration and invasion of HT-29 adenocarcinoma cells but supports their growth as tumors in nude mice. Cancer Res. 68 (23), 9746–9753. 10.1158/0008-5472.CAN-08-1321 19047153

[B5] Barbosa N. V. Nogueira C. W. Nogara P. A. de Bem A. F. Aschner M. Rocha J. B. T. (2017). Organoselenium compounds as mimics of selenoproteins and thiol modifier agents. Metallomics 9 (12), 1703–1734. 10.1039/c7mt00083a 29168872

[B6] Barrett C. W. Ning W. Chen X. Smith J. J. Washington M. K. Hill K. E. (2013). Tumor suppressor function of the plasma glutathione peroxidase gpx3 in colitis-associated carcinoma. Cancer Res. 73 (3), 1245–1255. Epub 2012 Dec 5. 10.1158/0008-5472.CAN-12-3150 23221387 PMC3563732

[B7] Bersuker K. Hendricks J. M. Li Z. Magtanong L. Ford B. Tang P. H. (2019). The CoQ oxidoreductase FSP1 acts parallel to GPX4 to inhibit ferroptosis. Nature 575 (7784), 688–692. Epub 2019 Oct 21. 10.1038/s41586-019-1705-2 31634900 PMC6883167

[B8] Bosello-Travain V. Conrad M. Cozza G. Negro A. Quartesan S. Rossetto M. (2013). Protein disulfide isomerase and glutathione are alternative substrates in the one Cys catalytic cycle of glutathione peroxidase 7. Biochim. Biophys. Acta 1830 (6), 3846–3857. Epub 2013 Feb 27. 10.1016/j.bbagen.2013.02.017 23454490

[B9] Bozinovski S. Seow H. J. Crack P. J. Anderson G. P. Vlahos R. (2012). Glutathione peroxidase-1 primes pro-inflammatory cytokine production after LPS challenge *in vivo* . PLoS One 7 (3), e33172. Epub 2012 Mar 6. 10.1371/journal.pone.0033172 22412999 PMC3295802

[B10] Brigelius-Flohé R. Kipp A. P. (2012). Physiological functions of GPx2 and its role in inflammation-triggered carcinogenesis. Ann. N. Y. Acad. Sci. 1259, 19–25. 10.1111/j.1749-6632.2012.06574.x 22758632

[B11] Brigelius-Flohé R. Maiorino M. (2013). Glutathione peroxidases. Biochim. Biophys. Acta 1830 (5), 3289–3303. Epub 2012 Nov 29. 10.1016/j.bbagen.2012.11.020 23201771

[B12] Brown C. W. Amante J. J. Chhoy P. Elaimy A. L. Liu H. Zhu L. J. (2019). Prominin2 drives ferroptosis resistance by stimulating iron export. Dev. Cell. 51 (5), 575–586. Epub 2019 Nov 14. 10.1016/j.devcel.2019.10.007 31735663 PMC8316835

[B13] Burk R. F. Olson G. E. Winfrey V. P. Hill K. E. Yin D. (2011). Glutathione peroxidase-3 produced by the kidney binds to a population of basement membranes in the gastrointestinal tract and in other tissues. Am. J. Physiol. Gastrointest. Liver Physiol. 301 (1), G32–G38. Epub 2011 Apr 14. 10.1152/ajpgi.00064.2011 21493731 PMC3280860

[B14] Chabory E. Damon C. Lenoir A. Kauselmann G. Kern H. Zevnik B. (2009). Epididymis seleno-independent glutathione peroxidase 5 maintains sperm DNA integrity in mice. J. Clin. Invest. 119 (7), 2074–2085. Epub 2009 Jun 22. 10.1172/JCI38940 19546506 PMC2701883

[B15] Chang C. Worley B. L. Phaëton R. Hempel N. (2020). Extracellular glutathione peroxidase GPx3 and its role in cancer. Cancers (Basel) 6, 12. 10.3390/cancers12082197 PMC7464599 32781581

[B16] Chen Y. I. Wei P. C. Hsu J. L. Su F. Y. Lee W. H. (2016). NPGPx (GPx7): A novel oxidative stress sensor/transmitter with multiple roles in redox homeostasis. Am. J. Transl. Res. 8 (4), 1626–1640. 27186289 PMC4859894

[B17] Ehrlich A. M. Pacheco A. R. Henrick B. M. Taft D. Xu G. Huda M. N. (2020). Indole-3-lactic acid associated with Bifidobacterium-dominated microbiota significantly decreases inflammation in intestinal epithelial cells. BMC Microbiol. 20 (1), 357. 10.1186/s12866-020-02023-y 33225894 PMC7681996

[B18] Esworthy R. S. Doroshow J. H. Chu F. F. (2022). The beginning of GPX2 and 30 years later. Free Radic. Biol. Med. 188, 419–433. Epub 2022 Jul 5. 10.1016/j.freeradbiomed.2022.06.232 35803440 PMC9341242

[B19] Flohé L. Toppo S. Orian L. (2022). The glutathione peroxidase family: Discoveries and mechanism. Free Radic. Biol. Med. 187, 113–122. Epub 2022 May 14. 10.1016/j.freeradbiomed.2022.05.003 35580774

[B20] Florian S. Wingler K. Schmehl K. Jacobasch G. Kreuzer O. J. Meyerhof W. (2001). Cellular and subcellular localization of gastrointestinal glutathione peroxidase in normal and malignant human intestinal tissue. Free Radic. Res. 35 (6), 655–663. 10.1080/10715760100301181 11811519

[B21] Forcina G. C. Dixon S. J. (2019). GPX4 at the crossroads of lipid homeostasis and ferroptosis. Proteomics 19 (18), e1800311. Epub 2019 May 31. 10.1002/pmic.201800311 30888116

[B22] Forrester S. J. Kikuchi D. S. Hernandes M. S. Xu Q. Griendling K. K. (2018). Reactive oxygen species in metabolic and inflammatory signaling. Circ. Res. 122 (6), 877–902. 10.1161/CIRCRESAHA.117.311401 29700084 PMC5926825

[B23] Gharagozloo P. Gutiérrez-Adán A. Champroux A. Noblanc A. Kocer A. Calle A. (2016). A novel antioxidant formulation designed to treat male infertility associated with oxidative stress: Promising preclinical evidence from animal models. Hum. Reprod. 31 (2), 252–262. 10.1093/humrep/dev302 26732620

[B24] Granatiero V. Konrad C. Bredvik K. Manfredi G. Kawamata H. (2019). Nrf2 signaling links ER oxidative protein folding and calcium homeostasis in health and disease. Life Sci. Alliance 2 (5), e201900563. 10.26508/lsa.201900563 31658977 PMC6819749

[B25] Gusti A. M. T. Qusti S. Y. Alshammari E. M. Toraih E. A. Fawzy M. S. (2021). Antioxidants-related superoxide dismutase (*SOD*), catalase (*cat*), glutathione peroxidase (*GPX*), glutathione-S-transferase (*gst*), and nitric oxide synthase (*NOS*) gene variants analysis in an obese population: A preliminary case-control study. Antioxidants (Basel) 10 (4), 595. 10.3390/antiox10040595 33924357 PMC8070436

[B26] Handy D. E. Joseph J. Loscalzo J. (2021). Selenium, a micronutrient that modulates cardiovascular health via redox enzymology. Nutrients 13 (9), 3238. 10.3390/nu13093238 34579115 PMC8471878

[B27] Handy D. E. Loscalzo J. (2022). The role of glutathione peroxidase-1 in health and disease. Free Radic. Biol. Med. 188, 146–161. Epub 2022 Jun 9. 10.1016/j.freeradbiomed.2022.06.004 35691509 PMC9586416

[B28] Ingold I. Berndt C. Schmitt S. Doll S. Poschmann G. Buday K. (2018). Selenium utilization by GPX4 is required to prevent hydroperoxide-induced ferroptosis. Cell. 172 (3), 409–422. Epub 2017 Dec 28. 10.1016/j.cell.2017.11.048 29290465

[B29] Jia M. Qin D. Zhao C. Chai L. Yu Z. Wang W. (2020). Redox homeostasis maintained by GPX4 facilitates STING activation. Nat. Immunol. 21 (7), 727–735. 10.1038/s41590-020-0699-0 32541831

[B30] Kanemura S. Sofia E. F. Hirai N. Okumura M. Kadokura H. Inaba K. (2020). Characterization of the endoplasmic reticulum-resident peroxidases GPx7 and GPx8 shows the higher oxidative activity of GPx7 and its linkage to oxidative protein folding. J. Biol. Chem. 295 (36), 12772–12785. Epub 2020 Jul 21. 10.1074/jbc.RA120.013607 32719007 PMC7476714

[B31] Kim H. J. Lee Y. Fang S. Kim W. Kim H. J. Kim J. W. (2020). GPx7 ameliorates non-alcoholic steatohepatitis by regulating oxidative stress. BMB Rep. 53 (6), 317–322. 10.5483/BMBRep.2020.53.6.280 32317079 PMC7330808

[B32] Kim J. E. Lee D. S. Kim T. H. Kang T. C. (2022). Glutathione regulates GPx1 expression during CA1 neuronal death and clasmatodendrosis in the rat Hippocampus following status epilepticus. Antioxidants (Basel) 11 (4), 756. 10.3390/antiox11040756 35453441 PMC9024994

[B33] Kim J. M. Kim H. G. Son C. G. (2018). Tissue-specific profiling of oxidative stress-associated transcriptome in a healthy mouse model. Int. J. Mol. Sci. 19 (10), 3174. 10.3390/ijms19103174 30326626 PMC6214011

[B34] Kipp A. P. Müller M. F. Göken E. M. Deubel S. Brigelius-Flohé R. (2012). The selenoproteins GPx2, TrxR2 and TrxR3 are regulated by Wnt signalling in the intestinal epithelium. Biochim. Biophys. Acta 1820 (10), 1588–1596. Epub 2012 Jun 7. 10.1016/j.bbagen.2012.05.016 22683372

[B35] Kipp A. P. (2017). Selenium-dependent glutathione peroxidases during tumor development. Adv. Cancer Res. 136, 109–138. Epub 2017 Aug 23. 10.1016/bs.acr.2017.07.004 29054415

[B36] Kraus R. J. Prohaska J. R. Ganther H. E. (1980). Oxidized forms of ovine erythrocyte glutathione peroxidase. Cyanide inhibition of a 4-glutathione:4-selenoenzyme. Biochim. Biophys. Acta 615 (1), 19–26. 10.1016/0005-2744(80)90004-2 7426660

[B37] Lahti P. P. Shariatmadari R. Penttinen J. K. Drevet J. R. Haendler B. Vierula M. (2001). Evaluation of the 5'-flanking regions of murine glutathione peroxidase five and cysteine-rich secretory protein-1 genes for directing transgene expression in mouse epididymis. Biol. Reprod. 64 (4), 1115–1121. 10.1095/biolreprod64.4.1115 11259257

[B38] Laribi A. Aouf S. Gabbouj S. Bouaouinaa N. Zakhama A. Harizi H. (2021). Human glutathione peroxidase codon 198 variant increases nasopharyngeal carcinoma risk and progression. Eur. Arch. Otorhinolaryngol. 278 (10), 4027–4034. Epub 2021 Feb 22. 10.1007/s00405-021-06628-5 33616746

[B39] Lee H. A. Chu K. B. Moon E. K. Quan F. S. (2021). Glutathione peroxidase 8 suppression by histone deacetylase inhibitors enhances endoplasmic reticulum stress and cell death by oxidative stress in hepatocellular carcinoma cells. Antioxidants (Basel) 10 (10), 1503. 10.3390/antiox10101503 34679638 PMC8533003

[B40] Lénon M. Ke N. Szady C. Sakhtah H. Ren G. Manta B. (2020). Improved production of Humira antibody in the genetically engineered *Escherichia coli* SHuffle, by co-expression of human PDI-GPx7 fusions. Appl. Microbiol. Biotechnol. 104 (22), 9693–9706. Epub 2020 Sep 30. 10.1007/s00253-020-10920-5 32997203 PMC7595990

[B41] Li F. Dai L. Niu J. (2020). GPX2 silencing relieves epithelial-mesenchymal transition, invasion, and metastasis in pancreatic cancer by downregulating Wnt pathway. J. Cell Physiol. 235 (11), 7780–7790. Epub 2019 Nov 27. 10.1002/jcp.29391 31774184

[B42] Li S. He Y. Chen K. Sun J. Zhang L. He Y. (2021). RSL3 drives ferroptosis through NF-κB pathway activation and GPX4 depletion in glioblastoma. Oxid. Med. Cell Longev. 2021, 2915019. 2021 Dec 26. 10.1155/2021/2915019 34987700 PMC8720588

[B43] Liu C. Yan Q. Gao C. Lin L. Wei J. (2021). Study on antioxidant effect of recombinant glutathione peroxidase 1. Int. J. Biol. Macromol. 170, 503–513. Epub 2020 Dec 28. 10.1016/j.ijbiomac.2020.12.183 33383079

[B44] Liu T. Sun L. Zhang Y. Wang Y. Zheng J. (2022). Imbalanced GSH/ROS and sequential cell death. J. Biochem. Mol. Toxicol. 36 (1), e22942. Epub 2021 Nov 2. 10.1002/jbt.22942 34725879

[B45] Lu B. Li J. Gui M. Yao L. Fan M. Zhou X. (2022). Salvianolic acid B inhibits myocardial I/R-induced ROS generation and cell apoptosis by regulating the TRIM8/GPX1 pathway. Pharm. Biol. 60 (1), 1458–1468. 10.1080/13880209.2022.2096644 35968584 PMC9380432

[B46] Luan Z. Fan X. Song H. Li R. Zhang W. Zhang J. (2019). Testosterone promotes GPX5 expression of goat epididymal epithelial cells cultured *in vitro* . Vitro Cell Dev. Biol. Anim. 55 (9), 677–685. Epub 2019 Aug 19. 10.1007/s11626-019-00391-y 31429037

[B47] Luan Z. Fan X. Zhao Y. Song H. Du W. Xu J. (2021). Trehalose can effectively protect sheep epididymis epithelial cells from oxidative stress. Arch. Anim. Breed. 64 (2), 335–343. 10.5194/aab-64-335-2021 34458560 PMC8386192

[B48] Lubos E. Mahoney C. E. Leopold J. A. Zhang Y. Y. Loscalzo J. Handy D. E. (2010). Glutathione peroxidase-1 modulates lipopolysaccharide-induced adhesion molecule expression in endothelial cells by altering CD14 expression. FASEB J. 24 (7), 2525–2532. Epub 2010 Mar 10. 10.1096/fj.09-147421 20219985 PMC2887263

[B49] Mamgain R. Kostic M. Singh F. V. (2022). Synthesis and antioxidant properties of organoselenium compounds. Curr. Med. Chem. Epub ahead of print. 10.2174/0929867329666220801165849 35927897

[B50] Mills G. C. (1957). Hemoglobin catabolism. I. Glutathione peroxidase, an erythrocyte enzyme which protects hemoglobin from oxidative breakdown. J. Biol. Chem. 229 (1), 189–197. 10.1016/s0021-9258(18)70608-x 13491573

[B51] Nguyen V. D. Saaranen M. J. Karala A. R. Lappi A. K. Wang L. Raykhel I. B. (2011). Two endoplasmic reticulum PDI peroxidases increase the efficiency of the use of peroxide during disulfide bond formation. J. Mol. Biol. 406 (3), 503–515. Epub 2011 Jan 5. 10.1016/j.jmb.2010.12.039 21215271

[B52] Nirgude S. Choudhary B. (2021). Insights into the role of GPX3, a highly efficient plasma antioxidant, in cancer. Biochem. Pharmacol. 184, 114365. Epub 2020 Dec 11. 10.1016/j.bcp.2020.114365 33310051

[B53] Noblanc A. Peltier M. Damon-Soubeyrand C. Kerchkove N. Chabory E. Vernet P. (2012). Epididymis response partly compensates for spermatozoa oxidative defects in snGPx4 and GPx5 double mutant mice. PLoS One 7 (6), e38565. Epub 2012 Jun 14. 10.1371/journal.pone.0038565 22719900 PMC3375294

[B54] Pérez S. Taléns-Visconti R. Rius-Pérez S. Finamor I. Sastre J. (2017). Redox signaling in the gastrointestinal tract. Free Radic. Biol. Med. 104, 75–103. Epub 2017 Jan 3. 10.1016/j.freeradbiomed.2016.12.048 28062361

[B55] Raykhel I. Alanen H. Salo K. Jurvansuu J. Nguyen V. D. Latva-Ranta M. (2007). A molecular specificity code for the three mammalian KDEL receptors. J. Cell Biol. 179 (6), 1193–1204. Erratum in: J Cell Biol. 2008 Feb 11;180(3):645. 10.1083/jcb.200705180 18086916 PMC2140024

[B56] Reddy A. T. Lakshmi S. P. Banno A. Reddy R. C. (2018). Role of GPx3 in PPARγ-induced protection against COPD-associated oxidative stress. Free Radic. Biol. Med. 126, 350–357. Epub 2018 Aug 15. 10.1016/j.freeradbiomed.2018.08.014 30118830 PMC6368849

[B57] Rusetskaya N. Y. Fedotov I. V. Koftina V. A. Borodulin V. B. (2019). Selenium compounds in redox regulation of inflammation and apoptosis. Biomed. Khim 65 (3), 165–179. Russian. 10.18097/PBMC20196503165 31258141

[B58] Seale L. A. Torres D. J. Berry M. J. Pitts M. W. (2020). A role for selenium-dependent GPX1 in SARS-CoV-2 virulence. Am. J. Clin. Nutr. 112 (2), 447–448. 10.1093/ajcn/nqaa177 32592394 PMC7337667

[B59] Seligman J. Newton G. L. Fahey R. C. Shalgi R. Kosower N. S. (2005). Nonprotein thiols and disulfides in rat epididymal spermatozoa and epididymal fluid: Role of gamma-glutamyl-transpeptidase in sperm maturation. J. Androl. 26 (5), 629–637. discussion 638-40. 10.2164/jandrol.05040 16088041

[B60] Strycharz-Dudziak M. Fołtyn S. Dworzański J. Kiełczykowska M. Malm M. Drop B. (2020). Glutathione peroxidase (GPx) and superoxide dismutase (SOD) in oropharyngeal cancer associated with EBV and HPV coinfection. Viruses 12 (9), 1008. 10.3390/v12091008 32917014 PMC7551554

[B61] Su L. Qu H. Cao Y. Zhu J. Zhang S. Z. Wu J. (2021). Effect of antioxidants on sperm quality parameters in subfertile men: A systematic review and network meta-analysis of randomized controlled trials. Adv. Nutr. 13 (2), 586–594. Epub ahead of print. 10.1093/advances/nmab127 34694345 PMC8970840

[B62] Tan S. Liu Q. Yang J. Cai J. Yu M. Ji Y. (2022). (MB) promotes oxidative stress-induced inhibiting of hepa1–6 cell proliferation via selenoprotein. Biol. Trace Elem. Res. Epub ahead of print. 10.1007/s12011-022-03120-x 35080709

[B63] Taylor A. Robson A. Houghton B. C. Jepson C. A. Ford W. C. Frayne J. (2013). Epididymal specific, selenium-independent GPX5 protects cells from oxidative stress-induced lipid peroxidation and DNA mutation. Hum. Reprod. 28 (9), 2332–2342. Epub 2013 May 21. 10.1093/humrep/det237 23696541

[B64] Tokuda E. Takei Y. I. Ohara S. Fujiwara N. Hozumi I. Furukawa Y. (2019). Wild-type Cu/Zn-superoxide dismutase is misfolded in cerebrospinal fluid of sporadic amyotrophic lateral sclerosis. Mol. Neurodegener. 14 (1), 42. 10.1186/s13024-019-0341-5 31744522 PMC6862823

[B65] Trenz T. S. Delaix C. L. Turchetto-Zolet A. C. Zamocky M. Lazzarotto F. Margis-PinheiroGoing Forward M. (2021). Going forward and back: The complex evolutionary history of the GPx. Biol. (Basel) 10 (11), 1165. 10.3390/biology10111165 PMC8614756 34827158

[B66] Tsuru-Aoyagi K. Potts M. B. Trivedi A. Pfankuch T. Raber J. Wendland M. (2009). Glutathione peroxidase activity modulates recovery in the injured immature brain. Ann. Neurol. 65 (5), 540–549. 10.1002/ana.21600 19475669 PMC2690612

[B67] Ursini F. Maiorino M. (2020). Lipid peroxidation and ferroptosis: The role of GSH and GPx4. Free Radic. Biol. Med. 152, 175–185. Epub 2020 Mar 9. 10.1016/j.freeradbiomed.2020.02.027 32165281

[B68] Usategui-Martín R. Puertas-Neyra K. Galindo-Cabello N. Hernández-Rodríguez L. A. González-Pérez F. Rodríguez-Cabello J. C. (2022). Retinal neuroprotective effect of mesenchymal stem cells secretome through modulation of oxidative stress, autophagy, and programmed cell death. Invest. Ophthalmol. Vis. Sci. 63 (4), 27. 10.1167/iovs.63.4.27 PMC9055551 35486068

[B69] Wang L. Zhang L. Niu Y. Sitia R. Wang C. C. (2014). Glutathione peroxidase 7 utilizes hydrogen peroxide generated by Ero1α to promote oxidative protein folding. Antioxid. Redox Signal 20 (4), 545–556. Epub 2013 Sep 17. 10.1089/ars.2013.5236 23919619 PMC3901321

[B70] Wang Q. Zhan S. Liu Y. Han F. Shi L. Han C. (2021). Low-Se diet can affect sperm quality and testicular glutathione peroxidase-4 activity in rats. Biol. Trace Elem. Res. 199 (10), 3752–3758. Epub 2021 Jan 7. 10.1007/s12011-020-02515-y 33415582

[B71] Wang X. Han Y. Chen F. Wang M. Xiao Y. Wang H. (2022). Glutathione peroxidase 1 protects against peroxynitrite-induced spiral ganglion neuron damage through attenuating NF-κB pathway activation. Front. Cell Neurosci. 16, 841731. 10.3389/fncel.2022.841731 35401119 PMC8983938

[B72] Wang Y. Cao P. Alshwmi M. Jiang N. Xiao Z. Jiang F. (2019). GPX2 suppression of H2O2 stress regulates cervical cancer metastasis and apoptosis via activation of the β-catenin-WNT pathway. Onco Targets Ther. 12, 6639–6651. 10.2147/OTT.S208781 31695405 PMC6707354

[B73] Wei P. C. Hsieh Y. H. Su M. I. Jiang X. Hsu P. H. Lo W. T. (2012). Loss of the oxidative stress sensor NPGPx compromises GRP78 chaperone activity and induces systemic disease. Mol. Cell. 48 (5), 747–759. Epub 2012 Nov 1. 10.1016/j.molcel.2012.10.007 23123197 PMC3582359

[B74] Wong C. H. Bozinovski S. Hertzog P. J. Hickey M. J. Crack P. J. (2008). Absence of glutathione peroxidase-1 exacerbates cerebral ischemia-reperfusion injury by reducing post-ischemic microvascular perfusion. J. Neurochem. 107 (1), 241–252. Epub 2008 Aug 7. 10.1111/j.1471-4159.2008.05605.x 18691391

[B75] Wu Y. Shi H. Xu Y. Pei J. Song S. Chen W. (2022). Ebselen ameliorates renal ischemia-reperfusion injury via enhancing autophagy in rats. Mol. Cell Biochem. 477 (6), 1873–1885. Epub 2022 Mar 26. 10.1007/s11010-022-04413-4 35338455

[B76] Xia Y. Pan W. Xiao X. Zhou X. Gu W. Liu Y. (2022). MicroRNA-483-5p accentuates cisplatin-induced acute kidney injury by targeting GPX3. Lab. Invest. 102 (6), 589–601. Epub 2022 Feb 18. 10.1038/s41374-022-00737-3 35184139

[B77] Xu S. Wu B. Zhong B. Lin L. Ding Y. Jin X. (2021). Naringenin alleviates myocardial ischemia/reperfusion injury by regulating the nuclear factor-erythroid factor 2-related factor 2 (Nrf2)/System xc-/glutathione peroxidase 4 (GPX4) axis to inhibit ferroptosis. Bioengineered 12 (2), 10924–10934. 10.1080/21655979.2021.1995994 34699317 PMC8809912

[B78] Yan W. Chen X. (2006). GPX2, a direct target of p63, inhibits oxidative stress-induced apoptosis in a p53-dependent manner. J. Biol. Chem. 281 (12), 7856–7862. Epub 2006 Jan 30. 10.1074/jbc.M512655200 16446369

[B79] Yang C. Guo X. Dong F. Meng F. Wang L. Wang P. (2022). miR-542-3p reduces antioxidant capacity in goat caput epididymal epithelial cells by targeting glutathione peroxidase 5 (GPx5). Theriogenology 186, 168–174. Epub 2022 Apr 20. 10.1016/j.theriogenology.2022.04.010 35487118

[B80] Yi Z. Jiang L. Zhao L. Zhou M. Ni Y. Yang Y. (2019). Glutathione peroxidase 3 (GPX3) suppresses the growth of melanoma cells through reactive oxygen species (ROS)-dependent stabilization of hypoxia-inducible factor 1-α and 2-α. J. Cell Biochem. 120 (11), 19124–19136. Epub 2019 Jul 16. 10.1002/jcb.29240 31310363

[B81] Yoboue E. D. Rimessi A. Anelli T. Pinton P. Sitia R. (2017). Regulation of calcium fluxes by GPX8, a type-II transmembrane peroxidase enriched at the mitochondria-associated endoplasmic reticulum membrane. Antioxid. Redox Signal 27 (9), 583–595. 10.1089/ars.2016.6866 28129698

[B82] Yu F. Yu C. Li F. Zuo Y. Wang Y. Yao L. (2021). Wnt/β-catenin signaling in cancers and targeted therapies. Signal Transduct. Target Ther. 6 (1), 307. 10.1038/s41392-021-00701-5 34456337 PMC8403677

[B83] Zhang T. Chabory E. Britan A. Grignard E. Pitiot O. Saez F. (2008). GPX5, the selenium-independent glutathione peroxidase-encoding single copy gene is differentially expressed in mouse epididymis. Reprod. Fertil. Dev. 20 (5), 615–625. 10.1071/rd08008 18577359

[B84] Zhao L. Zong W. Zhang H. Liu R. (2019). Kidney toxicity and response of selenium containing protein-glutathione peroxidase (Gpx3) to CdTe QDs on different levels. Toxicol. Sci. 168 (1), 201–208. 10.1093/toxsci/kfy297 30535317

[B85] Zmijewski J. W. Lorne E. Zhao X. Tsuruta Y. Sha Y. Liu G. (2009). Antiinflammatory effects of hydrogen peroxide in neutrophil activation and acute lung injury. Am. J. Respir. Crit. Care Med. 179 (8), 694–704. Epub 2009 Jan 16. 10.1164/rccm.200806-851OC 19151196 PMC2668804

